# Clinically Amyopathic Dermatomyositis Caused by a Tattoo

**DOI:** 10.1155/2018/7384681

**Published:** 2018-11-01

**Authors:** Bing Han, Qiang Guo

**Affiliations:** ^1^Department of Endocrinology, Shanghai Ninth People's Hospital, Shanghai Jiao Tong University School of Medicine, Shanghai, China; ^2^Department of Rheumatology, Renji Hospital, Shanghai Jiao Tong University School of Medicine, Shanghai, China

## Abstract

**Introduction:**

Clinically amyopathic dermatomyositis (CADM) is a rare disease with unknown origin. It is characterized by the specific skin lesions of dermatomyositis (DM) without clinical or laboratory evidence of myopathy. Previous studies indicated that tattoo may induce immune response.

**Case Report:**

A 22-year-old male who tattooed butterfly on the left chest with blue and red ink. Then, he gradually had typical Gottron rash and interstitial lung disease (ILD) without weakness of the muscle. The clinical presentation and laboratory test represent the diagnosis of CADM. According to the history, CADM was induced by the tattoo five months before admission.

**Discussion:**

We first reported the CADM induced by a tattoo. However, further studies are still needed to approach the specific substances within the tattoo that trigger immune response.

## 1. Introduction

Clinically amyopathic dermatomyositis (CADM) represents a subgroup of dermatomyositis (DM) patients who have typical skin manifestations but little evidence of myositis [1]. The incidence of CADM in all dermatomyositis patients is 5–20% [2, 3]. Tattoo ink is mainly comprised of pigments and carriers [4, 5]. With the popularity of tattooing, this practice also produces many problems such as infection, tattoo-associated dermatoses, and allergic reactions to tattoos.

We report a patient who had a butterfly tattooed on the right chest with blue and red ink. After receiving this tattoo, he gradually developed a typical Gottron rash and interstitial lung disease (ILD) without muscle weakness. The clinical presentation and laboratory test represent the diagnosis of CADM. There was no report of CADM related to the tattoo.

## 2. Case Report

**Figure 1:**
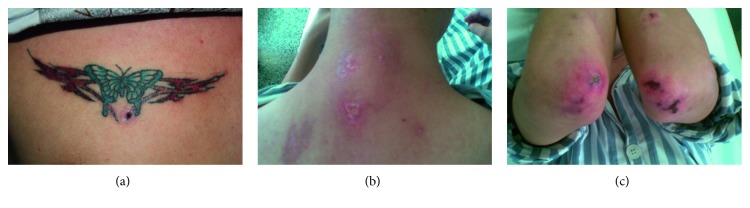
Clinical manifestation of the patient. (a) The patient with a butterfly tattooed on his chest. (b, c) Typical CADM rash in the back of neck and bilateral elbow.

**Figure 2:**
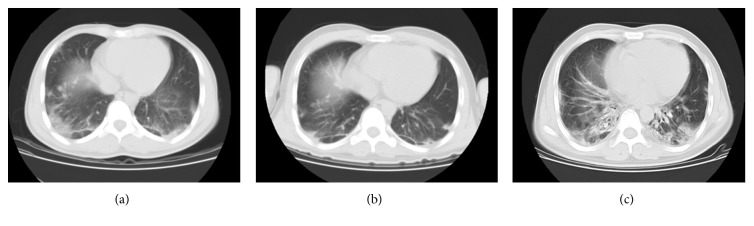
CT scan of patient in different stages. (a) A chest CT scan showing exudative lesions when he came to our hospital for the first time. (b) After treatment, the ILD gradually improved. (c) A CT scan indicating advanced ILD when the patient came to our hospital again.

**Figure 3:**
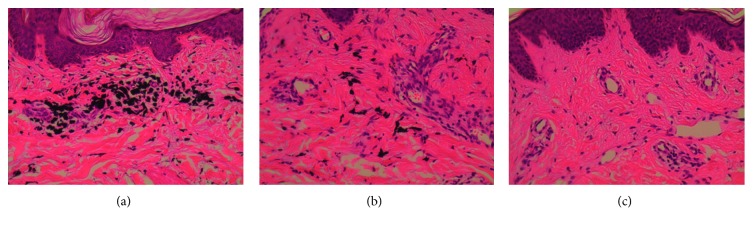
Dermatopathologic analysis of the patient. (a–c) Dermatopathologic analyses of the blue part and red part of the tattoo and of normal skin, respectively. However, there was no significant difference of lymphocytic infiltration between the blue and the red part of the tattoo.

A 22-year-old male presented at the hospital because of a rash, joint pain for four months, and breathlessness for one month. Five months before admission, he had tattooed a butterfly on his right chest with blue and red ink (Figure 1(a)). Then, four months before admission, erythema appeared on multiple parts of the skin, including the face, the extensor surface of the bilateral elbow, the metacarpophalangeal joints (MCP2–4), the neck, the chest, and the right side of the back (Figures 1(b) and 1(c)). However, there was no muscle weakness. Gradually, he began to develop shortness of breath after physical activity. A computed tomography (CT) scan of the chest indicated ILD (Figure 2(a)). Physical examination showed typical Gottron rash. C-reactive protein (CRP), erythrocyte sedimentation rate (ESR), rheumatoid factor (RF), electrolytes, glucose, hepatic/renal function, and hepatitis (A, B, and C) were all normal. Laboratory findings of antinuclear antibodies (ANA), extractable nuclear antigens (ENA), anti-centromere antibodies (ACA), complement (C3, C4, and CH50), immunoglobulin (IgM AG), antineutrophil cytoplasmic antibodies (ANCA), antinucleosome, cyclic citrullinated peptide (CCP) antibody, and glycoprotein I (GPI) were all within the normal range. He had normal creatine kinase levels (CK 32 U/L) and significantly increased levels of ferritin (1016.9 ng/ml). The above findings represent the diagnosis of CADM and ILD. He was treated with glucocorticoid and cyclosporin A (CsA). According to his history, we deduced that the CADM was caused by a tattoo in his right chest. So, the tattoo was surgically resected, and dermatopathologic analysis of the blue and red tattoo was performed with hematoxylin and eosin (HE) stain. There was no hyperplasia of the epidermis. Pigmentation associated with a small number of inflammatory cells and hyalinization of collagen fibers was detected in the superficial dermis. But no significant difference of lymphocytic infiltration was detected between the blue and the red part of the tattoo (Figures 3(a)–3(c)). After treatment, the rash and the ILD gradually improved (Figure 2(b)), and the patient was discharged from the hospital.

During the follow-up, he was admitted to our hospital again for shortness of breath after even minor activities a week after he was discharged. A CT scan indicated advanced ILD (Figure 2(c)). His blood routine test, electrolyte, CRP, ESR, liver function, and serum creatinine levels were within the normal range. T-SPOT and (1,3)-*β*-D glucan tests were also normal. The ferritin level was 888.6 ng/ml. He was treated with CsA and methylprednisolone. Amphotericin B, norvancomycin, cefoperazone-sulbactam, and SMZ were used to treat potential infections. Cyclophosphamide (CTX 0.4 g) and gamma globulin (10 g) were also intravenously injected. However, both the cytomegalovirus (CMV) and antibodies (IgG/IgM) were positive. So, ganciclovir was used to replace the amphotericin B and cefoperazone-sulbactam. Ten days later, the patient had a fever and felt breathless. Chest X-rays indicated diffused lesions in the bilateral lung. He was given BiPAP to assist ventilation. Imipenem-cilastatin, teicoplanin, and levofloxacin were used to control infection. Intubation was also conducted, as the oxygen saturation was continually lower than 80%. A lab test showed that white blood cell (WBC: 20.0 × 10^9^/L), ESR (53 mm/h), and CRP (52.9 mg/l) levels were obviously elevated. Blood gas indicated a low level of oxygen pressure (58.9 mmHg). Although the patient had a definite inducement, his respiratory failure gradually aggravated. Finally, he was dead of respiratory failure.

## 3. Discussion

CADM is a rare disease with unknown origin. It is characterized by the specific skin lesions of DM without clinical or laboratory evidence of myopathy. ILD is the major cause of mortality in ADM patients. It can accompany both DM and CADM, but rapidly progressive ILD (RPILD) is more common in patients with CADM [6]. In a retrospective study of 41 Chinese CADM patients, Sun et al. [7] reported that the prevalence of ILD was 60.98%, including 26.83% for acute/subacute interstitial pneumonia (A/SIP) and 34.15% for chronic interstitial pneumonia (CIP). They also reported the mortality of A/SIP as 63.64%, with a 6-month survival rate of 54.50%. However, there is currently no consensus on the treatment of CADM [8]. Li et al. [9] showed that pirfenidone may improve the prognosis of patients with subacute ILD related to CADM.

Researchers reported that CADM was associated with various tumors. Nogi et al. [10] even reported that recession of the tumor may improve interstitial pneumonia and cutaneous manifestations. The CADM patients with rapidly progressive ILD had specific autoantibodies, originally called anti-melanoma differentiation-associated gene 5 (MDA5) antibody [11]. Muro et al. [12] found that environmental factors contribute to the production of this antibody, which initiates innate antiviral responses. Our patient tattooed a butterfly on his chest and suffered CADM a month later; the ink of the tattoo may have contained some kind of substance that induced an immune reaction.

Tattoo ink is mainly comprised of two components: pigments and carriers [4, 5]. As the popularity of tattooing continues to rise, it has been noted to cause various medical problems. Case reports of infection, tattoo-associated dermatoses, and allergic reactions to tattoos are frequently reported in the literature. [4] In some cases, tattoos may induce a Koebner reaction in some dermatologic conditions. Additionally, Jolly [13] described a case of discoid lupus erythematosus developing after tattooing. Abreu Velez et al. [14] found macrophage markers (HAM56 and CD68) surrounding the tattoo pigment, which indicated interaction of the immune system with tattoo components. Since the literature concerning the immune response to tattoos is not common, further investigation of the immune response to tattoos is merited.

## 4. Conclusion

In summary, we reported a case of CADM related to a tattoo in one of our patients. However, further studies are needed to determine the specific substances within the tattoo that triggered the immune response.
